# Longitudinal health‐related quality of life in first‐line treated patients with chronic lymphocytic leukemia: Results from the Connect^®^ CLL Registry

**DOI:** 10.1002/jha2.67

**Published:** 2020-07-26

**Authors:** Jeff P. Sharman, Kim Cocks, Chadi Nabhan, Nicole Lamanna, Neil E. Kay, David L. Grinblatt, Christopher R. Flowers, Matthew S. Davids, Pavel Kiselev, Arlene S. Swern, Kristen Sullivan, Mecide M. Gharibo, E. Dawn Flick, Andrew Trigg, Anthony Mato

**Affiliations:** ^1^ Willamette Valley Cancer Institute US Oncology Eugene Oregon USA; ^2^ Adelphi Values Bollington Cheshire UK; ^3^ Caris Life Sciences Dallas Texas USA; ^4^ University of South Carolina Columbia South Carolina USA; ^5^ Division of Hematology and Oncology Department of Medicine New York‐Presbyterian/Columbia University Medical Center New York New York USA; ^6^ Division of Hematology Mayo Clinic Rochester Minnesota USA; ^7^ NorthShore University HealthSystem Evanston Illinois USA; ^8^ MD Anderson Cancer Center University of Texas Houston Texas USA; ^9^ Department of Medical Oncology Dana‐Farber Cancer Institute Boston Massachusetts USA; ^10^ Bristol Myers Squibb Princeton New Jersey USA; ^11^ Memorial Sloan Kettering Cancer Center New York New York USA

**Keywords:** chronic lymphocytic leukemia, first‐line therapy, lymphocytes, quality of life, registry

## Abstract

Health‐related quality of life (HRQoL) in patients with chronic lymphocytic leukemia (CLL) is important in guiding treatment decisions. However, the impact of CLL treatment initiation on HRQoL is unclear. We assessed HRQoL using the FACT‐Leu and EQ‐5D‐3L questionnaires in the Connect ***^®^*** CLL Registry, a large, US‐based, multicenter, prospective observational study of CLL patients enrolled between 2010 and 2014, prior to the introduction of novel therapies. Among 889 patients initiating first‐line therapy with chemoimmunotherapy or rituximab monotherapy, questionnaire completion rates were 95.7% and 95.8% at enrollment, and 70.8% and 69.4% at 12 months, for FACT‐Leu Total and EQ‐5D‐3L, respectively. For 849 patients completing all five FACT‐Leu components, average total scores were 135.7 at enrollment and 141.6 at 12 months. Among 526 patients with FACT‐Leu Total scores at enrollment and 12 months, clinically meaningful (≥11‐point) improvements or reductions were observed in 179 (34.0%) and 88 (16.7%) patients, respectively. Mean EQ‐5D‐3L index scores were 0.87 at enrollment and 12 months. Among 513 patients completing EQ‐5D‐3L at enrollment and 12 months, clinically meaningful (≥0.06‐point) improvements or reductions were observed in 125 (24.4%) and 116 (22.6%) patients, respectively. In the Connect^®^ CLL Registry, HRQoL remained stable or slightly improved after 12 months of follow‐up.

## 1 INTRODUCTION

Chronic lymphocytic leukemia (CLL) is generally considered incurable, even with the increasing availability of novel treatments [1]. Most patients experience a chronic disease course with periods of relapse and remission, for which they usually receive multiple lines of therapy. Current guidelines recommend treatment initiation only in symptomatic patients, for both first‐line therapy (LOT1) and subsequent lines of therapy [1, 2]

Patients’ health‐related quality of life (HRQoL) plays an important role in guiding treatment decisions and assessing the impact of cancer treatment [3]. Previous studies on HRQoL in patients with CLL treated with chemoimmunotherapy (CIT) have mostly focused on global health status and fatigue, which have often been measured using cancer‐generic HRQoL instruments, including the European Organization for Research and Treatment of Cancer (EORTC) 30‐item quality of life questionnaire (QLQ‐C30) [4, 5, 6, 7, 8], rather than more sensitive leukemia‐specific instruments such as the Functional Assessment of Cancer Therapy–Leukemia (FACT‐Leu), which may more accurately represent the symptoms experienced by patients with CLL [9, 10, 11]. Longitudinal data on HRQoL in CLL are also sparse [12] and have mostly been reported in observational studies with small sample sizes [13, 14].

In this study from the Connect ***^®^*** CLL Registry, longitudinal analyses of HRQoL were performed in a predominantly community‐based cohort of patients with CLL, focusing on those patients undergoing LOT1 with CIT or rituximab (R) monotherapy at enrollment into the Registry. As the Registry was initiated prior to the approval of novel agents such as the Bruton tyrosine kinase inhibitors, phosphoinositide 3‐kinase inhibitors, and venetoclax, no patients received these agents during LOT1. Patients receiving LOT1 were included in the analysis to avoid introducing additional variability caused by prior therapies and disease progression that may confound the analyses. HRQoL was assessed using the FACT‐Leu and the EuroQol five‐dimensional three‐level (EQ‐5D‐3L) questionnaires, instruments that are easy to complete, widely used, and validated in CLL and other cancers [9, 10, 11, 12, 15, 16, 17]. To understand whether changes in HRQoL scores were clinically meaningful, the minimally important difference (MID), that is the smallest change in an outcome that a patient would identify as important or that would result in a change in treatment, was utilized [18].

## 2 METHODS

### 2.1 The Connect^®^ CLL registry

The Connect^®^ CLL Registry (NCT01081015) is a large, US‐based, multicenter, prospective observational cohort study of adult patients with CLL. Full details on the Registry design have been described previously [19]. Further details of the Registry are provided in the Supporting Information Methods.

### 2.2 HRQoL assessments

HRQoL was assessed using the FACT‐Leu and EQ‐5D‐3L questionnaires (see Supporting Information Methods for further details). Briefly, the FACT‐Leu consists of five components: Physical, Social, Emotional, and Functional Well‐Being, plus leukemia‐specific Additional Concerns [9, 10]. FACT‐Leu Total scores are presented for patients who completed ≥36 of 44 items overall, regardless of completion of all the individual components [9, 20]. Component scores are presented for patients who answered ≥50% of items for each of the five FACT‐Leu components. Higher scores across all domains denote better HRQoL.

The EQ‐5D‐3L consists of five domains covering mobility, self‐care, usual activities, pain/discomfort, and anxiety/depression [21]. Results are reported as individual domain scores and as a summary index score. Patients also reported their self‐rated health on a visual analogue scale (VAS). Lower scores on the EQ‐5D‐3L domains and higher scores on the EQ‐5D‐3L index and VAS correspond to better HRQoL.

### 2.3 Statistical analysis

The study closed 31 December, 2017. Mean HRQoL changes from baseline and differences between patient clusters over time were analyzed using a repeated measures regression model. To determine whether changes in HRQoL scores were clinically meaningful, the MID for each instrument was prespecified and defined in accordance with previously published limits [22, 23]. For the FACT‐Leu Total scores, the MID was 11 [23]. For the EQ‐5D‐3L index‐based scores, the MID was 0.06 [22].

Univariate and multivariable logistic regression analyses were performed to identify factors associated with clinically meaningful improvements in HRQoL on the FACT‐Leu Total scores and EQ‐5D‐3L index scores from baseline to 12 months (see Supporting Information Methods). Variables with a χ^2^ *P*‐value < .1 in the univariate analyses were included in the multivariable model.

Comparisons of categorical variables were performed using the χ^2^ test; continuous variables were compared using the Wilcoxon rank‐sum test. Two‐sided tests at *P *< .05 were considered statistically significant. Statistical analyses were performed using SAS software, version 9.2 (SAS Institute, Cary, NC, USA).

## 3 RESULTS

### 3.1 Patient characteristics

From March 2010 to January 2014, 1494 patients were enrolled in the Registry from 199 centers throughout the United States: 179 community (n = 1311), 17 academic (n = 155), and three government centers (n = 28). Of these patients, 889 were enrolled in LOT1. Baseline patient and treatment characteristics are shown in Table 1. Median time from CLL diagnosis to Registry enrollment was 1.5 years (range 0‐32).

**TABLE 1:** Baseline and treatment characteristics in patients receiving LOT1

Characteristic^*^	Completed FACT‐Leu and EQ‐5D‐3L at baseline N = 828	Overall N = 889
Age, median (range), years	68 (22‐99)	68 (22‐99)
Male sex, n (%)	530 (64.0)	566 (63.7)
Race, n (%)		
White	747 (90.2)	798 (89.8)
African‐American	53 (6.4)	56 (6.3)
Asian	1 (0.1)	3 (0.3)
Other	5 (0.6)	6 (0.7)
Not reported	22 (2.7)	26 (2.9)
US geographic region, n (%)		
South	335 (40.5)	352 (39.6)
Midwest	256 (30.9)	277 (31.2)
West	128 (15.5)	140 (15.7)
Northeast	102 (12.3)	112 (12.6)
Not reported	7 (0.8)	8 (0.9)
ECOG PS, n (%)		
0	329 (39.7)	347 (39.0)
1	272 (32.9)	296 (33.3)
≥2	43 (5.2)	47 (5.3)
Not reported	184 (22.2)	199 (22.4)
CCI score		
Median (range)	2.0 (2.0‐10.0)	2.0 (2.0‐10.0)
≤2, n (%)	475 (57.4)	511 (57.5)
≥3, n (%)	353 (42.6)	378 (42.5)
Duration between CLL diagnosis and Registry enrollment, median (range), years	1.5 (0–32)	1.5 (0–32)
Rai stage, n (%)		
0	154 (18.6)	172 (19.3)
I	177 (21.4)	191 (21.5)
II	101 (12.2)	108 (12.1)
III	103 (12.4)	107 (12.0)
IV	101 (12.2)	107 (12.0)
Not reported	192 (23.2)	204 (22.9)
Constitutional symptoms, n (%)		
Fatigue	444 (53.6)	480 (54.0)
Night sweats	213 (25.7)	226 (25.4)
Weight loss	151 (18.2)	157 (17.7)
Fever	58 (7.0)	60 (6.7)
Other	102 (12.3)	113 (12.7)
Not reported	291 (35.1)	309 (34.8)
Institution type, n (%)		
Academic	80 (9.7)	86 (9.7)
Community	733 (88.5)	787 (88.5)
Government	15 (1.8)	16 (1.8)
Treatment at enrollment, n (%)		
FCR	233 (28.1)	246 (27.7)
BR	183 (22.1)	199 (22.4)
R monotherapy	96 (11.6)	103 (11.6)
Other	316 (38.2)	341 (38.4)

Abbreviations: BR, bendamustine and rituximab; CCI, Charlson Comorbidity Index; CLL, chronic lymphocytic leukemia; ECOG PS, Eastern Cooperative Oncology Group performance status; EQ‐5D‐3L, EuroQol 5‐dimension 3‐level questionnaire; FACT‐Leu, Functional Assessment of Cancer Therapy–Leukemia; FCR, fludarabine, cyclophosphamide, and rituximab; R, rituximab; LOT1, first‐line therapy.

* Rounding may cause totals to be < or > 100%.

### 3.2 HRQoL assessments

**FIGURE 1:**
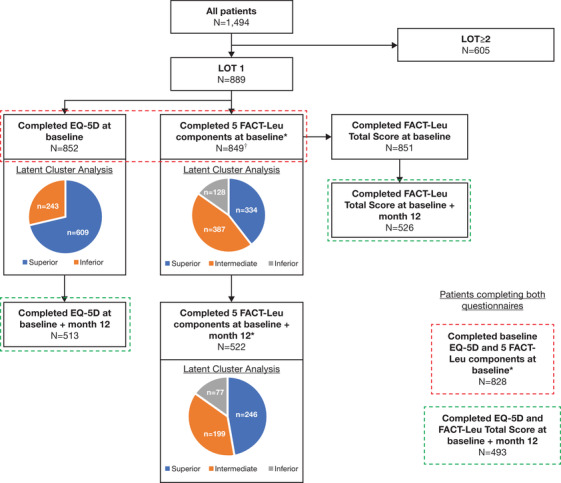
Study flow chart. *FACT‐Leu component scores were only reported if patients answered ≥4 questions on the Physical, Social, Emotional, and Functional Well‐Being scales; and ≥9 questions on the Additional Concerns scale. ^†^Of 849 patients completing the five FACT‐Leu components at baseline, 524 also completed the FACT‐Leu Total score at 12 months. Abbreviations: EQ‐5D‐3L, EuroQol 5‐dimension 3‐level questionnaire; FACT‐Leu, Functional Assessment of Cancer Therapy–Leukemia; LOT, line of treatment

FACT‐Leu Total completion rates in the LOT1 cohort varied between 95.7% (n = 851) at baseline and 70.8% (n = 546) at 12 months of follow‐up (Table 2). A total of 526 patients provided FACT‐Leu Total scores at baseline and 12 months, but did not necessarily complete all five components; 522 patients provided ≥50% responses for all five FACT‐Leu components at baseline and at 12 months (Figure 1). EQ‐5D‐3L completion rates decreased from 95.8% (n = 852) at baseline to 69.4% (n = 535) at 12 months of follow‐up. In all, 513 patients completed the EQ‐5D‐3L questionnaire at both time points. In the LOT1 cohort, 849 patients completed the five FACT‐Leu components at baseline, 852 patients completed the EQ‐5D‐3L at baseline, and 828 patients completed both the FACT‐Leu and EQ‐5D‐3L at baseline (Figure 1). Factors associated with non‐completion of HRQoL questionnaires are described in the Supporting Information Results.

**TABLE 2:** HRQoL questionnaire completion rates in 889 patients receiving LOT1

Visit	Patients in the Registry, N	Completed both HRQoL forms, n (%)	Completed FACT‐Leu Total, n (%)	Completed EQ‐5D‐3L index, n (%)
Baseline	889	830 (93.4)	851 (95.7)	852 (95.8)
3 months	864	708 (81.9)	729 (84.4)	720 (83.3)
6 months	839	601 (71.6)	614 (73.2)	615 (73.3)
9 months	799	574 (71.8)	585 (73.2)	584 (73.1)
12 months	771	529 (68.6)	546 (70.8)	535 (69.4)

Abbreviations: EQ‐5D‐3L, EuroQol 5‐dimension 3‐level questionnaire; FACT‐Leu, Functional Assessment of Cancer Therapy–Leukemia; HRQoL, health‐related quality of life; LOT1, first‐line therapy.

In the 828 patients who completed both questionnaires at baseline, baseline characteristics were similar to those from the full LOT1 cohort (Table 1).

### 3.3 FACT‐Leu

**FIGURE 2:**
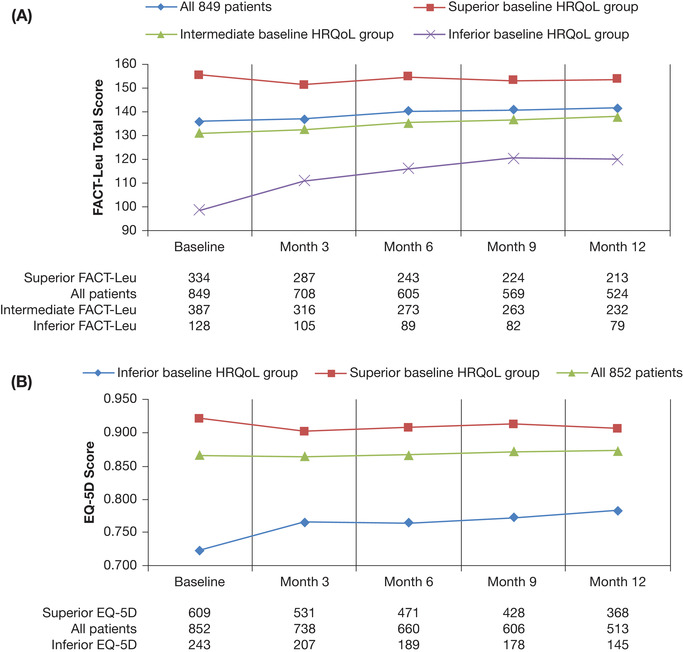
Changes in total HRQoL scores over time in patients with CLL receiving LOT1 overall and by baseline HRQoL group as measured by (A) FACT‐Leu Total scores and (B) EQ‐5D‐3L index. A, Higher FACT‐Leu Total scores indicate better HRQoL. “All patients” refers to 849 patients completing the FACT‐Leu Total score at baseline. B, Higher EQ‐5D‐3L index scores indicate better HRQoL. “All patients” refers to 852 patients completing the EQ‐5D‐3L at baseline. Abbreviations: CLL, chronic lymphocytic leukemia; EQ‐5D‐3L, EuroQol 5‐dimension 3‐level questionnaire; FACT‐Leu, Functional Assessment of Cancer Therapy–Leukemia; HRQoL: health‐related quality of life; LOT1, first‐line therapy. In a subset analysis of 522 patients completing the FACT‐Leu components at baseline and at 12 months, similar results were seen for patients with inferior, intermediate, and superior HRQoL scores at baseline (data not shown)

**FIGURE 3:**
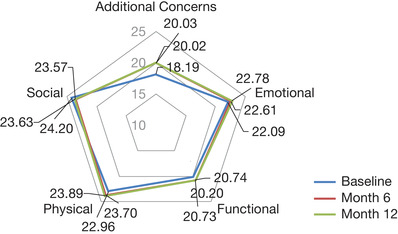
Changes in FACT‐Leu scores between baseline and 12 months in 522 patients with CLL receiving LOT1. “Additional Concerns” and “Emotional Well‐Being” scores were rescaled to match the other categories (0‐28). Emotional Well‐Being subscale was rescaled using the following equation: floor (Emotional Well‐Being score × 28/24). Additional Concerns subscale was rescaled using the following equation: floor([Additional Concerns score ‐ 14] × 28/54). Higher scores on the Physical and Emotional Well‐Being domains and the items covering Additional Concerns correspond to worse HRQoL, whereas higher scores on the Social and Functional Well‐Being domains reflect better HRQoL. Abbreviations: CLL, chronic lymphocytic leukemia; FACT‐Leu, Functional Assessment of Cancer Therapy–Leukemia; HRQoL, health‐related quality of life; LOT1, first‐line therapy

Among the 849 patients who completed all five FACT‐Leu components at baseline, FACT‐Leu Total scores were 135.7 at baseline and 141.6 at 12 months for the 524 patients who also completed the FACT‐Leu Total score at 12 months (Figure 2A). A total of 526 patients provided FACT‐Leu Total scores at baseline and at 12 months, of whom 179 (34.0%) had a clinically meaningful (≥11‐point) improvement in FACT‐Leu Total scores, while 88 (16.7%) had a clinically meaningful (≥11‐point) reduction. Mean scores remained relatively stable between baseline and 12 months for Physical (23.0‐23.9), Social (23.6‐24.2), Emotional (22.1‐22.8), and Functional Well‐Being (20.2‐20.7) (Table 3; Figure 3). These changes were all within the MID of 2‐3 points specified for these domains [23]. The most impacted FACT‐Leu domain was the Additional Concerns (eg, constitutional symptoms, weakness, and lumps or swelling; Table 3; Figure 3). The mean Additional Concerns scores were 18.2 at baseline, 20.0 at 6 months, and 20.0 at 12 months.

**TABLE 3:** Mean FACT‐Leu scores at baseline and at 6 and 12 months by baseline FACT‐Leu cluster in patients with CLL receiving LOT1

FACT‐Leu subscale	Inferior baseline FACT‐Leu N = 77			Intermediate baseline FACT‐Leu N = 199			Superior baseline FACT‐Leu N = 246			Overall N = 522		
	Baseline	6 months	12 months	Baseline	6 months	12 months	Baseline	6 months	12 months	Baseline	6 months	12 months
Physical	17.3	20.4	21.0	21.6	22.6	23.0	25.8	25.5	25.5	23.0	23.7	23.9
Social	20.8	20.4	21.6	23.4	23.1	22.9	25·9	24·9	24·9	24·2	23·6	23·6
Functional	12·3	15·3	16·5	18·3	19·4	19·3	24·2	23·5	23·3	20·2	20·7	20·7
Emotional*	17·6	19·5	20·3	21·3	21·8	21·8	24·1	24·2	24·3	22·1	22·6	22·8
Additional Concerns*	11·6	16·5	16·5	16·2	18·3	18·7	21·9	22·5	22·2	18·2	20·0	20·0

Abbreviations: CLL, chronic lymphocytic leukemia; FACT‐Leu, Functional Assessment of Cancer Therapy–Leukemia; HRQoL, health‐related quality of life; LOT1, first‐line therapy.

*In order to present all scores on the spider plots, scores were rescaled to match the other categories (0‐28). Emotional subscale was rescaled using the following equation: floor(Emotional Well‐Being score × 28/24). Additional Concerns subscale was rescaled using the following equation: floor([Additional Concerns score − 14] × 28/54). Higher scores reflect better HRQoL.

In the 849 patients who completed all five FACT‐Leu components at baseline, seven Additional Concerns of interest were analyzed (Figure S1); fever, chills, fatigue, weight loss, night sweats, weakness, and lumps or swelling. The highest mean scores at baseline, indicating worse HRQoL, were observed for fatigue (1.8 [n = 842]) and weakness (1.2 [n = 844]). At 12 months, the corresponding mean scores were 1.5 (n = 524) for fatigue and 0.9 (n = 523) for weakness. While scores were lower at 12 months for both Additional Concerns, indicating an improvement in HRQoL, these changes were below the threshold for a clinically meaningful change (MID of 5.1‐6.8 for Additional Concerns) [23]. The largest decline in mean scores between baseline and 12 months, representing an improvement in Additional Concerns, was seen for lumps or swelling (–0.5), weight loss (–0.5), and night sweats (–0.3); however, these changes were not clinically meaningful.

To identify subgroups of patients with similar changes in HRQoL over time, latent cluster analysis was utilized based on HRQoL scores at baseline (Supporting Information Methods). The 849 patients who completed all five FACT‐Leu components at baseline were clustered into three groups: inferior, intermediate, and superior clusters. There were 334 patients in the superior FACT‐Leu cluster (39.3%), 387 in the intermediate FACT‐Leu cluster (45.6%), and 128 in the inferior FACT‐Leu cluster (15.1%) (Figure 1). Differences between baseline characteristics of patients in each cluster are described in the Supporting Information Results.

**FIGURE 4:**
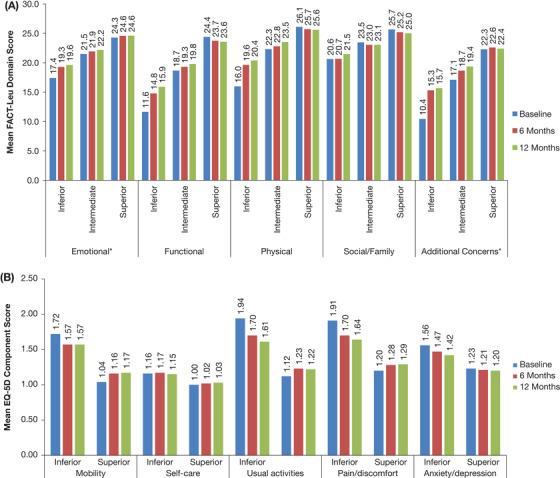
Mean change in scores over time in patients with CLL receiving LOT1 by (A) baseline FACT‐Leu cluster (849 patients) and (B) baseline EQ‐5D‐3L cluster (852 patients). A, *Emotional Well‐Being and Additional Concerns domains were rescaled to the range 0‐28, in order to present the domains on the same chart. B, Lower EQ‐5D component scores indicate better HRQoL. Abbreviations: CLL, chronic lymphocytic leukemia; EQ‐5D‐3L, EuroQol 5‐dimension 3‐level questionnaire; FACT‐Leu, Functional Assessment of Cancer Therapy–Leukemia. HRQoL, health‐related quality of life; LOT1, first‐line therapy

There was a trend toward increased FACT‐Leu scores from baseline to 12 months in the inferior group for the FACT‐Leu Total Score (Figure 2A) and across all domains (Figure 4A) while scores remained stable for patients in the superior group.

### 3.4 EQ‐5D‐3L

Among 852 patients who completed the EQ‐5D‐3L at baseline, mean EQ‐5D‐3L index scores were 0.87 at baseline and 0.87 at 12 months (Figure 2B). Of the 513 patients who completed the EQ‐5D‐3L at baseline and at 12 months, 125 (24.4%) had a clinically meaningful (≥0.06‐point) improvement in EQ‐5D‐3L index scores and 116 (22.6%) had a clinically meaningful (≥0.06‐point) reduction in EQ‐5D‐3L index scores.

In the 852 patients who completed baseline EQ‐5D‐3L questionnaires, patients were clustered into two groups based on baseline EQ‐5D‐3L scores; inferior and superior (Figure 1). EQ‐5D‐3L scores by cluster are shown in Figure 2B. The superior EQ‐5D‐3L cluster consisted of 609 patients (71.5%), with a mean EQ‐5D‐3L index score of 0.92 over the first 12 months. The inferior EQ‐5D‐3L cluster included 243 patients (28.5%) whose mean EQ‐5D‐3L index score was 0.72. Differences between baseline characteristics of patients in each cluster are described in the Supporting Information Results.

Cluster analyses showed differences in HRQoL between the clusters across most EQ‐5D‐3L components except self‐care (Figure 4B). In the inferior EQ‐5D‐3L cluster, there was a trend toward improved EQ‐5D‐3L domain and index scores in the first 3‐6 months, with smaller improvements seen in months 6‐12 on most scores except self‐care. In the superior EQ‐5D‐3L cluster, there was a trend toward worsened EQ‐5D‐3L component and index scores in the first 3‐6 months, with stable scores seen thereafter.

### 3.5 Treatment received and HRQoL

**FIGURE 5:**
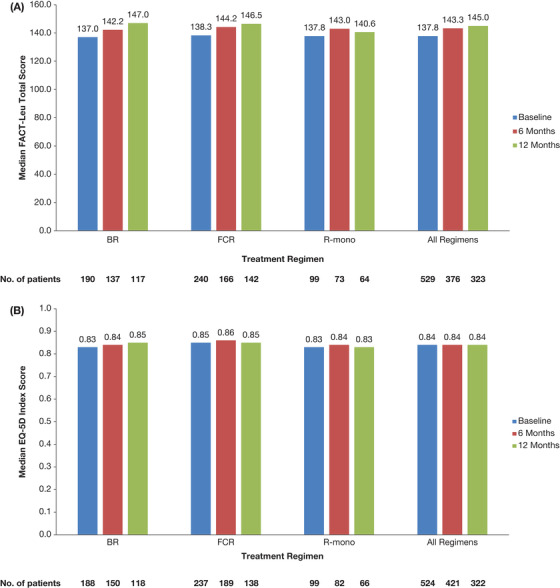
Median changes in HRQoL scores over time in patients with CLL receiving LOT1 by treatment regimen for (A) FACT‐Leu Total scores and (B) EQ‐5D‐3L index. A, Higher FACT‐Leu total scores indicate better HRQoL. B, Higher EQ‐5D index scores indicate better HRQoL. Abbreviations: BR, bendamustine and rituximab; CLL, chronic lymphocytic leukemia; EQ‐5D‐3L, EuroQol 5‐dimension 3‐level questionnaire; FACT‐Leu, Functional Assessment of Cancer Therapy–Leukemia; FCR, fludarabine, cyclophosphamide, and rituximab; HRQoL, health‐related quality of life; LOT1, first‐line therapy; R‐mono, rituximab monotherapy

Of the 851 patients completing FACT‐Leu Total scores at baseline, 240 (28.2%) patients received fludarabine, cyclophosphamide, and rituximab (FCR), 190 (22.3%) received bendamustine and rituximab (BR), and 99 (11.6%) received rituximab (R) monotherapy as the first line of therapy. In patients treated with BR or FCR, there was a trend toward improved median FACT‐Leu Total scores at 12 months. (Figure 5A).

For the 852 patients completing EQ‐5D‐3L questionnaires at baseline, 246 (28.9%) patients received FCR, 199 (23.4%) received BR, and 103 (12.1%) received R monotherapy, with a similar distribution between EQ‐5D‐3L clusters. Patients who received FCR were more likely to have better baseline HRQoL. In patients treated with BR, there was a trend toward improved median EQ‐5D‐3L component and index scores over time, while the scores remained stable at 12 months in the FCR group (Figure 5B).

### 3.6 Predictors of HRQoL improvement

A total of 493 patients provided both EQ‐5D‐3L index and FACT‐Leu Total score at both the baseline and the 12‐month time point. Of these, 74 (15.0%) patients had clinically meaningful improvements in both EQ‐5D‐3L index and FACT‐Leu Total score over 12 months. Multivariable analyses showed that Eastern Cooperative Oncology Group performance status (ECOG PS) 0‐1 versus ≥2 (*P =* .021), absence of del(17p) (*P =* .049), and treatment with FCR versus other therapies (*P =* .024) were associated with clinically meaningful HRQoL improvements with both questionnaires at 12 months (data not shown).

## 4 DISCUSSION

At 12 months of follow‐up after enrollment in the Connect^®^ CLL Registry, first‐line treatment appears to result in positive changes in HRQoL with HRQoL measurements remaining stable or improving compared with baseline scores. While more patients experienced a clinically meaningful improvement in FACT‐Leu Total scores than a clinically meaningful reduction (34.3% vs 16.8%), clinically meaningful improvement and reduction in EQ‐5D‐3L scores was similar (24.4% vs 22.6%). This may be due to the leukemia‐specific nature of the FACT‐Leu instrument that may more closely reflect patients’ symptoms and experience of their disease. Furthermore, the trend toward an improvement in HRQoL, as measured by FACT‐Leu, in FCR‐treated patients may be due to a greater sensitivity in detecting leukemia‐specific improvements in HRQoL with this leukemia‐focused instrument. The Additional Concerns items, such as “lumps or swelling,” which would likely be treatment‐sensitive, showed the greatest improvement in the FACT‐Leu score.

Data regarding the effect of treatment initiation in CLL on HRQoL are mixed. In the GCLLSG CLL8 trial, no significant differences in HRQoL were observed between fludarabine and cyclophosphamide (FC) and FCR during treatment or follow‐up as measured by the EORTC QLQ‐C30 questionnaire, despite a higher adverse event burden with FCR [6]. Similarly, in the GCLLSG CLL4 trial there was no difference in HRQoL, as assessed by EORTC QLQ‐C30, between fludarabine monotherapy and FC [24]. Two trials of lenalidomide and ofatumumab showed either no effect or a slight positive effect of treatment on HRQoL, respectively [15, 25]. However, in two population‐based studies from the Netherlands, initiation of chemotherapy or CIT was associated with a considerable worsening of HRQoL [14, 26]. Differences in setting, study design, and population should be considered when interpreting these data. Furthermore, the long‐term effect of treatment must not be discounted. EORTC QLQ‐C30 data comparing chlorambucil monotherapy, fludarabine monotherapy, and FC showed initial differences in HRQoL between patients receiving fludarabine and those on chlorambucil alone. However, these differences were transient and all patients experienced a positive effect on HRQoL when remission was ultimately achieved [27]. These factors may also explain why there was no significant improvement in HRQoL among patients receiving first‐line treatment in the Connect CLL Registry. All patients in the Registry received chemotherapy regimens at LOT1, which are known to have a high adverse event burden [28] and require hospital visits in order to receive the drug infusions. These factors may negatively affect the patient perception of their health or the impact of the disease on their daily life. However, all patients were receiving first‐line treatment for CLL, reducing confounding caused by prior therapies and patients with relapsed/refractory disease. Therefore, these findings provide important insight into the real‐world experiences of patients undergoing first‐line treatment with chemotherapy regimens.

Our study has several limitations. Selection bias can occur as physicians may select certain patients for enrollment. Additionally, median time between CLL diagnosis and Registry enrollment, for which treatment initiation is a requirement, was relatively short in the Registry patients compared with the overall CLL population [29], suggesting a possible bias toward a higher‐risk population. To minimize these limitations, consecutive patients presenting to the sites were evaluated for enrollment and invited to participate. However, the observed improvement in mean HRQoL scores over time may have been influenced by early patient dropout, that is patients with poorer health dropping out of the Registry or declining further HRQoL participation earlier than healthier patients, which could bias the mean score upward. Subset sensitivity analyses showed that patients with lower ECOG PS scores, indicating better health, were more likely to complete both questionnaires at 12 months. However, baseline HRQoL scores themselves were not associated with 12‐month completion rates. Additionally, the repeated measures regression model applied is robust to data missing not at random. Treatment at academic centers, younger patient age, and non‐white race were all associated with non‐compliance with HRQoL questionnaires. This may indicate an unmet need for greater patient education to encourage questionnaire completion or to identify those patients requiring more support to complete questionnaires.

Due to the timing of the Connect^®^ CLL Registry, no patients received first‐line treatment with novel agents. Novel agents such as ibrutinib, idelalisib, and venetoclax are now commonly used following approval by the US Food and Drug Administration for previously untreated and relapsed/refractory patients. A recent analysis of HRQoL in the HELIOS study showed that there was no change in HRQoL between patients with relapsed CLL receiving ibrutinib plus BR (followed by ibrutinib alone) versus placebo plus BR (followed by ibrutinib alone). However, the subset of patients who had worse well‐being, physical functioning, and fatigue at baseline experienced greater improvements in these HRQoL outcomes with ibrutinib plus BR versus placebo plus BR [4]. Similarly, in the COMPLEMENT2 trial, the addition of the anti‐CD20 antibody ofatumumab to FC did not affect HRQoL, as measured by the EORTC QLQ‐C30 and EORTC Quality of Life Questionnaire – CLL module, while small improvements in HRQoL were maintained after treatment [7].

As with any HRQoL study, it is important to consider whether statistically significant differences are relevant and meaningful for patients. While a MID threshold of 0.06 is generally used for EQ‐5D‐3L index scores in US patients [22], no clear MID thresholds have been defined for the individual EQ‐5D‐3L domains. Our data also suggest that the leukemia‐specific FACT‐Leu measure may be more sensitive at detecting incremental HRQoL changes following initiation of a new treatment. While the EQ‐5D‐3L instrument has been used in other studies of CLL [4, 14, 15, 16], patients may have felt that the generic EQ‐5D‐3L questionnaire did not accurately capture their experiences of living with CLL, as reflected in the lower EQ‐5D‐3L completion rates. The FACT‐Leu questionnaire is a relatively new HRQoL instrument, which has been validated in adult patients with acute and chronic leukemia [9]. Few studies have reported the use of the FACT‐Leu in patients with CLL [12, 15, 16], but it has been used in patients with chronic myeloid leukemia [30, 31, 32, 33, 34] and acute myeloid leukemia [35, 36]. Increased use of this measure in real‐world and clinical trial settings will help define its utility and the impact of treatment on HRQoL in patients with CLL.

In the Connect^®^ CLL Registry in which patients were predominantly treated with CIT in the first‐line, HRQoL remained stable to slightly improved over 12 months of follow‐up. No significant increases in HRQoL were observed, mirroring previous reports in which treatment initiation did not have a significant impact on HRQoL. It will be important to assess these indices in the era of novel targeted therapies and to validate how FACT‐Leu and EQ‐5D‐3L values in CLL compare with other chronic medical conditions.

## AUTHOR CONTRIBUTION

J.P.S., C.N., N.L., N.E.K., D.L.G., C.R.F., M.S.D., and A.M. enrolled patients to the Registry. P.K. and A.S.S. completed the statistical analyses; and all authors interpreted the data, directed the development, review, and approval of this manuscript, and are fully responsible for all content and editorial decisions.

## DATA SHARING STATEMENT

Data requests may be submitted to Celgene, A Bristol‐Myers Squibb Company at https://vivli.org/ourmember/celgene/ and must include a description of the research proposal.

## CONFLICT OF INTEREST

J.P.S. has received honoraria/research funding from Abbvie, Acerta, Celgene Corporation, Genentech, Gilead, Janssen, Pharmacyclics and TG Therapeutics. K.C. is employed by Adelphi Values; has received consulting fees from Bristol Myers Squibb, formerly Celgene Corporation, for this work; and has received statistical consulting fees from Amgen and Endomag Ltd. C.N. is employed by Cardinal Health Specialty Solutions; has received research funding from Astellas, Celgene Corporation, Genentech and Seattle Genetics; and has been on advisory boards for AbbVie, Celgene Corporation, Cardinal Health, Genentech and Infinity. N.L. has received research funding from AbbVie, AstraZeneca, Beigene, Genentech, Gilead, Infinity, Juno, Ming, Oncternal, Pronai and TG Therapeutics; has been a consultant for AbbVie, AstraZeneca, Beigene, Genentech, Gilead, Jannsen, Pharmacyclics and Pronai; and has been on an advisory committee for Celgene Corporation. N.E.K. has received research funding from Celgene Corporation, Genentech, Gilead, Hospira and Pharmacyclics; and has been on advisory committees for Celgene Corporation and Gilead. D.L.G. has been a consultant and member of a speakers’ bureau for Celgene Corporation. C.R.F. has received research funding from AbbVie, Acerta, Gilead, Infinity, Janssen, Millennium, Pharmacyclics, Spectrum and TG Therapeutics; has been a consultant for Celgene Corporation, Genentech/Roche, Gilead, Millennium, Optum Rx and Seattle Genetics; and has received honoraria from Celgene Corporation. M.S.D. has served as a consultant for AbbVie, Acerta Pharma, Adaptive Biotechnologies, Ascentage Pharma, AstraZeneca, Beigene, Genentech, Janssen, MEI Pharma, Pharmacyclics, Research to Practice, Sunesis, Syros Pharmaceuticals, TG Therapeutics and Verastem; and received research funding from Acerta Pharma, Ascentage Pharma, Genentech, MEI Pharma, Pharmacyclics, Surface Oncology, TG Therapeutics and Verastem. P.K., A.S.S., K.S., M.M.G. and E.D.F. are employees of Bristol Myers Squibb and have equity. A.T. is employed by Adelphi Values, an agency that consults with various pharmaceutical companies. A.M. has received research funding from AbbVie, Celgene Corporation, Gilead, Pronai and TG Therapeutics; has been a consultant for AbbVie; and has been a member of a speakers’ bureau for Celgene Corporation.

## Supporting information

Fig S1. Study flow chart.
Fig S2. Changes in FACT‐Leu scores between baseline and 12 months in 522 patients with CLL receiving LOT1.
Fig S3. Mean FACT‐Leu Additional Concerns of Interest* scores between baseline and 12 months in 849 patients with CLL receiving LOT1 who completed FACT‐Leu at baseline.
