# Supplementary material for: Longitudinal health‐related quality of life in first‐line treated patients with chronic lymphocytic leukemia: Results from the Connect® CLL Registry
Source: EJHaem. 2020 Jul 26;1(1):188–98. doi: 10.1002/jha2.67 (PMC9176138; doi:10.1002/jha2.67)
Supplement: Supplementary file 1 — Fig S1. Study flow chart. Fig S2. Changes in FACT‐Leu scores between baseline and 12 months in 522 patients with CLL receiving LOT1. Fig S3. Mean FACT‐Leu Additional Concerns of Interest* scores between baseline and 12 months in 849 patients with CLL receiving LOT1 who completed FACT‐Leu at baseline. [file JHA2-1-188-s001.docx]

**Supporting Information**

**Supplemental Methods**

***The Connect^®^*** ***CLL Registry***

The Connect^®^ CLL Registry (Mato *et al*, 2016) included study centres with an adequate number of patients with CLL and experience in registries or haematology/oncology trials. Eligible patients had CLL as defined by the International Workshop on CLL (Hallek *et al*, 2008) and were enrolled in the Registry between March 2010 and January 2014; “baseline” was defined as the time at which patients provided informed consent. Sites were invited to enrol all eligible patients consecutively as they visited their haematologist. Each centre could enrol up to 30 patients. Patients initiating a new line of therapy ≤60 days prior to study enrolment were eligible for inclusion. The Connect^®^ CLL Registry is non-interventional, with all medical care performed solely at the discretion of the treating clinician in accordance with standard clinical practice at each site. All participants provided written informed consent. The study was conducted in accordance with the Declaration of Helsinki. The registry protocol was approved by a central institutional review board (IRB) (Quorum Review IRB, Seattle, WA, USA) or each site’s respective IRB. Patient data were recorded in an electronic data capture system at baseline and every 3 months, for up to 5 years or until early discontinuation (due to study termination, patient withdrawal, loss to follow-up, or death). This study is reported in line with the Strengthening the Reporting of Observational Studies in Epidemiology (STROBE) guidelines for reporting of observational studies (STROBE checklist v4).

For the purpose of this analysis, only patients initiating LOT1 were included. These analyses were deemed the most reliable, as outcomes are not affected by earlier treatments, the patient population is more homogeneous, and fewer confounding factors are present compared with patients receiving LOT≥2.

***HRQoL assessments – FACT-Leu***

Sites were asked to administer paper HRQoL questionnaires at the time of informed consent and at each physician visit during routine clinic visits throughout follow-up (approximately once every 3 months) for up to 5 years. The FACT-Leu is a reliable and efficient HRQoL instrument that has been validated in patients with acute and chronic leukaemia (Cella *et al*, 2012). The questionnaire consists of 5 components: the cancer-specific FACT-General (FACT-G) base questionnaire, which includes 27 items covering 4 domains (Physical, Social, Emotional, and Functional Well-Being) plus the 17-item Leukemia subscale covering leukaemia-specific Additional Concerns, e.g. constitutional symptoms, weakness, and lumps or swelling (Cella *et* *al*, 1993; Cella *et al*, 2012). Patients score each item on a 5-point Likert scale, with 0 = not at all and 4 = very much.

***HRQoL assessments – EQ-5D-3L***

The EQ-5D-3L consists of 5 domains covering mobility, self-care, usual activities, pain/discomfort, and anxiety/depression. The EQ-5D-3L is a standardized instrument that is used to quantitatively measure HRQoL in a wide range of diseases (EuroQol Group). Its 5 domains (mobility, self-care, usual activities, pain/discomfort, and anxiety/depression) are scored on a scale of 1–3, with 1 signifying no problems and 3 corresponding to extreme problems. Results are reported as individual domain scores and as a summary index score, which ranges from −0·109 to 1 (Shaw *et al*, 2005). Patients report their self-rated health on a VAS, ranging from 0 to 100. Questionnaires are considered complete provided that no more than 20% of the questions have missing responses.

***Statistical analysis – cluster analysis***

Clusters of similar patients were identified through latent cluster analysis based on HRQoL scores at baseline. Patients with the lowest HRQoL scores were grouped in the inferior, cluster, patients with intermediate scores in an intermediate, cluster, and patients with the highest scores at baseline were grouped in the and superior cluster. Patients were assigned to clusters based on their highest predicted probability of membership using Akaike and Bayesian information criteria. Spider plots were used to assess and visualize changes in individual components of the HRQoL instruments. In order to present all scales on the same spider plot, the FACT-Leu scores for Emotional Well-Being (0–24) and Additional Concerns (14–68) were rescaled to match the range of scores for the other domains (0–28).

***Univariable and multivariate logistic regression***

The following characteristics were included in the analyses: study site (academic vs. other), sex (male vs. female), age (<75 vs. ≥75 years), CD38 (positive vs. negative/unknown), ECOG PS (0–1 vs. ≥2), del(17p) (absent vs. present), region (Midwest vs. Northeast vs. South vs. West), treatment (FCR vs. BR vs. R monotherapy] vs. other), prior malignancies (no vs. yes), Charlson Comorbidity Index (CCI) score (≤2 vs. ≥3), Rai stage (0–1 vs. ≥2), insurance (private vs. other/unknown), and race (white vs. other/unknown). Variables with a χ^2^ *P* value <0·1 in the univariate analyses were included in the multivariable model. To further explore the impact of treatment regimen on HRQoL, treatment was grouped to provide a binary comparison (FCR vs. other) in the 12-month multivariable model.

***Missing data***

As per Registry protocol, missing data were not imputed and were assumed to be missing at random. To investigate the patterns associated with missing HRQoL data at 12 months, univariate analyses were performed comparing baseline demographic and disease characteristics for those patients who completed the HRQoL questionnaires at month 12 with those who did not complete the questionnaires at month 12.

**Supplemental Results**

***Factors associated with non-completion of HRQoL questionnaires***

A total of 873 patients completed at least 1 HRQoL assessment at baseline, either the FACT-Leu or EQ-5D-3L questionnaire. HRQoL data at 12 months were missing for 330 patients (37·8%) of whom 115 (34·8%) discontinued the study prior to completing 12 months of follow-up. Baseline characteristics for the remaining 215 patients who did not complete 12-month HRQoL assessments were compared with those of 543 patients who completed assessments at 12 months.

Baseline HRQoL scores did not influence completion of 12-month assessments: mean differences between 12 month completers and non-completers in both FACT-Leu Total score (136·7 vs. 134·2) and EQ-5D-3L index (0·87 vs. 0·84) were not statistically significant. There was, however, an association between 12-month completion rates and baseline characteristics in multivariable analysis, including race (white 73% vs. non-white 61%), age (>75 years 77% vs. ≤75 years 70%), and institution type (academic 51% vs. community/government 74%). Also, those who received BR or FCR as the first regimen were more likely to complete 12-month HRQoL assessments than those who received other therapies.

***Differences in baseline characteristics of patients in each FACT-Leu cluster***

When comparing the baseline characteristics of patients in the different FACT-Leu clusters, compared with the superior and intermediate FACT-Leu clusters, patients in the inferior FACT-Leu cluster were more often female (30·5% vs. 38·0% vs. 45·3%) and more likely to be insured with Medicare/Medicaid (59·3% vs. 62·8% vs. 72·7%). Age was generally similar between groups (median age 68·0 vs. 67·0 vs. 68·0 years). Patients in the intermediate and inferior FACT-Leu clusters were more likely to have constitutional symptoms (75·2% vs. 79·7%) than patients in the superior FACT-Leu cluster (49·1%). The most frequent reason for initiating treatment in the superior FACT-Leu group was the number of regions with enlarged nodes/progressive/symptomatic lymphadenopathy (38·3%). In the intermediate FACT-Leu group progressive marrow failure was the most common reason for treatment initiation (41·3%), while it was disease-related symptoms (46·1%) (including fatigue [32·8%], night sweats [26·6%], weight loss [14·1%], and fever [7·0%]) in patients in the inferior FACT-Leu group (data not shown).

***Differences in baseline characteristics of patients in each EQ-5D-3L cluster***

When comparing the baseline characteristics of patients in the different EQ-5D-3L clusters, patients in the inferior EQ-5D-3L cluster were generally older (mean age 70·2 vs. 66·5 years), more often female (40·3% vs. 34·2%), more often insured via Medicare/Medicaid (74·5% vs. 57·2%), and more frequently had constitutional symptoms (80·5% vs. 60·0%) compared with the superior EQ-5D-3L cluster. In the inferior EQ-5D-3L cluster, the most frequent reason for treatment initiation was disease-related symptoms (43·2%) (including fatigue [30·5%], night sweats [23·5%], weight loss [13·2%], and fever [4·1%]), followed by progressive marrow failure (40·7%). In the superior EQ-5D-3L cluster, progressive marrow failure (39·7%) was the most frequent reason given, followed by number of regions with enlarged nodes/progressive/symptomatic lymphadenopathy (36·0%) (data not shown).


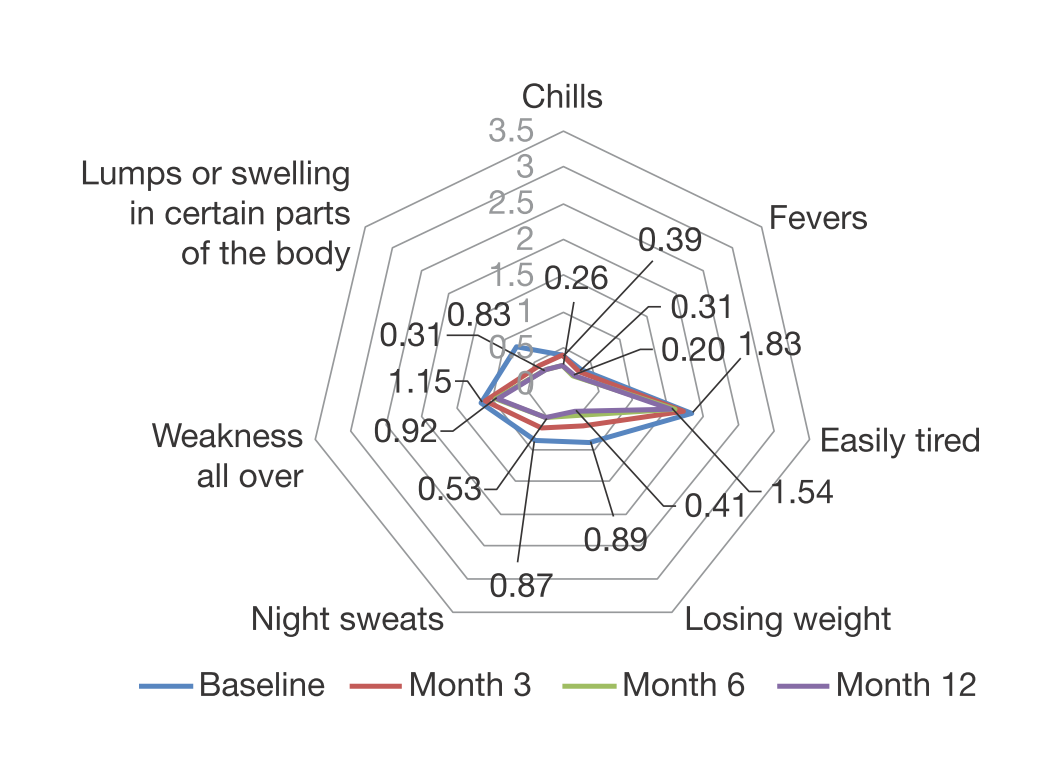


**Fig S1.** Mean FACT-Leu Additional Concerns of Interest* scores between baseline and 12 months in 849 patients with CLL receiving LOT1 who completed FACT-Leu at baseline. Lower scores on the FACT-Leu Additional Concerns scales indicate better HRQoL. * Additional Concerns of Interest: BRM2, I am bothered by the chills; BRM3, I am bothered by fevers, BMT6, I get tired easily; C2, I am losing weight; ES3, I have night sweats; HI12, I feel weak all over; LEU1, I am bothered by lumps or swelling in certain parts of my body. CLL, chronic lymphocytic leukaemia; FACT-Leu, Functional Assessment of Cancer Therapy–Leukemia; LOT1, first-line therapy.
